# Second Virial Coefficients for N_2_···H_2_ and NH···NH

**DOI:** 10.1021/acs.jpca.5c04624

**Published:** 2026-01-02

**Authors:** Marcos D. S. Alves, Maikel Y. Ballester

**Affiliations:** Departamento de Física, 28113 Universidade Federal de Juiz de Fora, Juiz de Fora, Minas Gerais CEP 36036-900, Brazil

## Abstract

Thermodynamic properties of real gases can be accurately described using realistic intermolecular potential energy surfaces. In this work, a first-order correction to the ideal gas equation of state is introduced through the computation of the classical second virial coefficient, *B*(*T*), derived from the configurational partition function, which explicitly depends on the intermolecular interaction potential. As a case study, the double many-body expansion (DMBE) potential energy surface for the ground electronic state of the N_2_H_2_ system was employed to derive pairwise interaction potentials for H_2_···N_2_ and NH···NH. These potentials were used to numerically evaluate the canonical partition function. Second virial coefficients, compressibility factors, and constant-volume heat capacities were computed in the temperature range 30–2000 K. The calculated *B*(*T*) values for H_2_···N_2_ are in good agreement with previous literature data, while the results for NH···NH lie within expected trends observed for similar systems.

## Graphical abstract

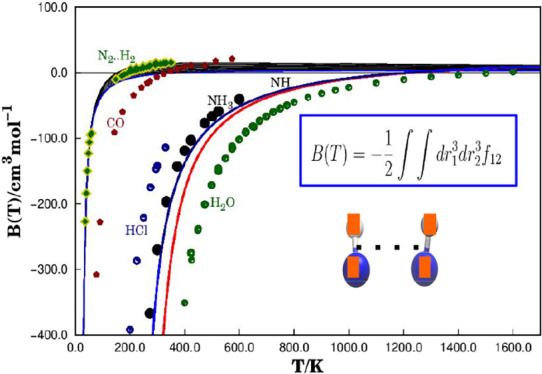

## Introduction

Deviations from ideal behavior are among the most intriguing features of real gases. Within the framework of statistical mechanics, intermolecular interactions play a fundamental role in determining the thermophysical properties of dilute gases. These interactions are described by potential energy surfaces (PES), which provide detailed information about the forces that govern the interactions between particles. Based on a given PES, the behavior of gases in the low-density limit can be analyzed by computing the second virial coefficient.^1–4^

Thermodynamic quantities such as the compressibility factor (*Z*), heat capacities (*C*_p_, *C*_v_), internal energy, enthalpy, and entropy can be derived from the virial coefficients by combining ideal and residual gas contributions.^5,6^ However, calculating the second virial coefficient as a function of temperature is far from trivial.^5–8^ Representing a multidimensional interaction potential analytically is particularly challenging, especially when ensuring correct physical behavior at both short and long ranges.^9–11^ Additionally, the multidimensional integrals required over the full configuration space often necessitate efficient sampling strategies,^4^ and the choice of coordinate systems can further complicate the description of atomic configurations. These difficulties have perhaps limited the number of studies in the literature dedicated to report virial coefficients derived from high-level, fully analytic potentials.

The interaction between two NH radicals, although typically representing a secondary process, plays a crucial role in high-temperature environments, such as combustion systems, due to the high reactivity of NH species.^12^ This reaction pathway contributes to the depletion of NH and the formation of nitrogen-containing intermediates, with implications for NO_*x*_ production cycles.^13^ A detailed kinetic understanding—supported by transition-state theory and master-equation simulations—is essential to model NH_2_OH thermal decomposition and, more broadly, to inform strategies for combustion optimization and pollutant control.

In this study, we employ the analytic PES for the ground electronic state of N_2_H_2_, developed by Poveda et al.,^11^ to calculate the cross second virial coefficient associated with N_2_···H_2_ and NH···NH. This PES has been thoroughly validated through both computational and experimental benchmarks, demonstrating excellent accuracy in representing interaction potentials. To the best of our knowledge, the second virial coefficient for the NH dimer has not yet been reported in the literature, highlighting the novelty of the present work. As a first-order correction to the ideal gas law, our approach enables improved modeling of real-gas behavior. We further explore thermodynamic properties relevant to the Joule–Thomson process, with results benchmarked against literature data for N_2_···H_2_ to assess the performance and applicability of the method.

## Methodology

### Potential Energy Surface

The potential energy surface (PES) adopted in this study was constructed by Poveda, Biczysko, and Varandas in the frame of the double many-body expansion (DMBE) approach.^11^ This formulation ensures the correct description of dissociation limits from physically motivated functions, and reproduces short-range interactions mimicking high-level ab initio data. The DMBE PES provides a global analytic representation of the N_2_H_2_ ground electronic state for all the configurations of the tetratomic system. This PES has been employed in molecular dynamics studies involving collisions of its fragments.^14–16^

Such a function was developed in terms of the six interatomic distances of the four atoms: $V_{N_{2}H_{2}}(\vec{R})$ ; $\vec{R}\equiv (R_{N_{\left(1\right)}N_{\left(2\right)}},R_{N_{\left(1\right)}H_{\left(1\right)}},R_{N_{\left(1\right)}H_{\left(2\right)}},R_{N_{\left(2\right)}H_{\left(1\right)}},R_{N_{\left(2\right)}H_{\left(2\right)}},R_{H_{\left(1\right)}H_{\left(2\right)}})$ . For the interest of this work, a dimensionality reduction was first implemented as

$$V_{N_{2}H_{2}}(\vec{R})\to u_{12}(r_{12},\theta_{1},\theta_{2},\phi )$$ 1

adopting the diatom–diatom Jacobi coordinate system to represent the fully anisotropic potential between two rigid diatoms,^17^ as illustrated in Figure 1. Within this framework, the Mayer function is given by

$$f_{12}(r_{12},\theta_{1},\theta_{2},\phi )=\exp[-\beta u_{12}(r_{12},\theta_{1},\theta_{2},\phi )]-1$$ 2

where β = 1/*k*_B_ *T*, and *k*_B_ is the Boltzmann constant. Further details on the coordinate transformation are provided in Section S.1 of the Supporting Material.

**Figure 1:**
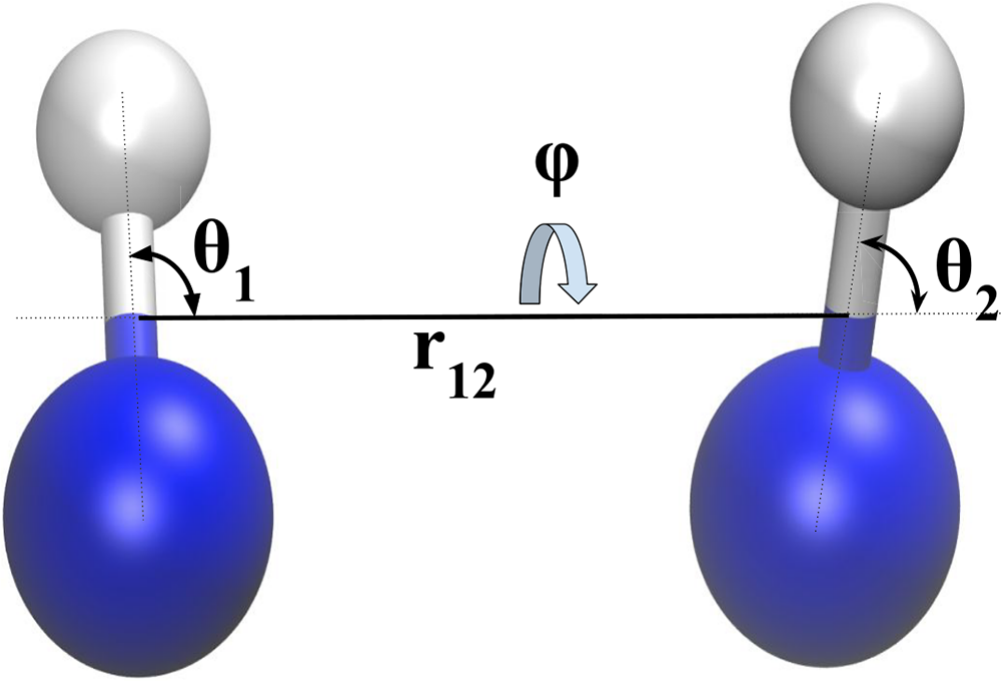
Jacobi coordinates for the diatom–diatom system.

To illustrate the potential profile, one-dimensional cuts are displayed in panel (a) of the Figure 2. Selected angular values were fixed, while the intermolecular distance changed. These plots show representative effective potentials that capture the anisotropy of the NH–NH interactions under specific angular configurations. For some of these configurations, the corresponding Mayer function for the NH dimers was also calculated, and is presented in panel (b) of Figure 2. The interaction energy is expressed in Hartree (*E*_h_) units, and all distances in Bohrs (*a*_0_ = 0.529 × 10^–10^ m).

**Figure 2:**
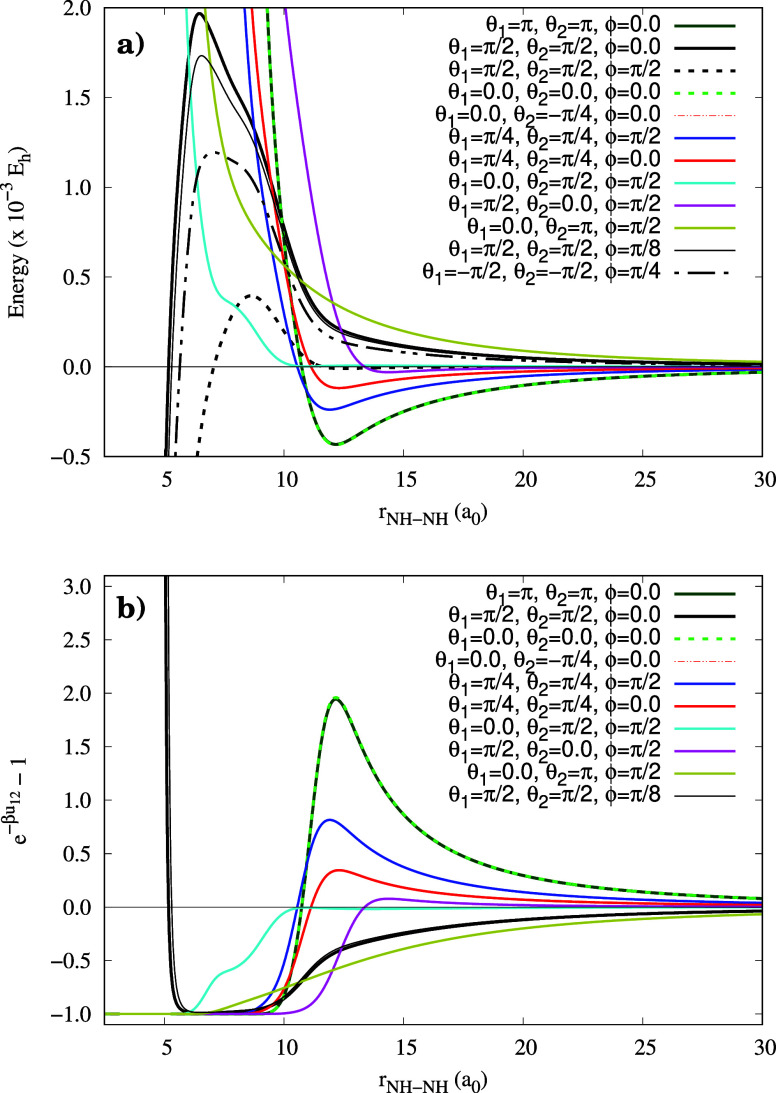
(a) NH–NH interaction potential for different orientations in Jacobi coordinates. (b) Corresponding Mayer functions for the same configurations.

The potential profiles in Figure 2 for some orientations exhibit the typical behavior of a van der Waals (vdW) potential energy curve. In turn, for other configurations, such as the designated “H” (θ_1_ = θ_2_ = π/2 for the NH–NH dimer), a barrier-like potential is observed before reaching the short-range region. Such low energy at small distances is responsible for the formation of the chemical bond (H–N=N–H).

This highly anisotropic characteristic is crucial for calculating the virial coefficient: in these specific geometries, the Mayer function *f*(*r*) diverges due to the deep potential well. Yet, the divergent range strongly depends upon the selected orientation.

### Virial Equation of State

The virial expansion for a real gas takes the form:^1,2,18–20^

$$Z=\frac{p}{\rho_{n}RT}=1+B\rho_{n}+C\rho_{n}^{2}+D\rho_{n}^{3}+...$$ 3

where ρ_n_ = *n*/*V* is the molar density. Alternatively, in terms of pressure:

$$Z=1+\tilde{B}p+\tilde{C}p^{2}+\tilde{D}p^{3}+...$$ 4

with the relations:

$$B=\tilde{B}RT,\;C\sim =\frac{C-B^{2}}{(RT{)}^{2}}$$

In eqs 3 and 4, the virial coefficients *B*, *C*, *D* describe deviations from the behavior of the ideal gas. The first term represents the contribution of the ideal gas. The coefficients are temperature dependent and reflect the nature of intermolecular interactions: *B*(*T*) corresponds to pair interactions, *C*(*T*) to terms of three bodies, etc. This formulation constitutes a cluster expansion, derived from the grand canonical ensemble by expressing pressure and density as power series in fugacity. At the thermodynamic limit (*V* → *∞*, *N* → *∞*), the expansion yields the virial equation of state. Consequently, an accurate description of intermolecular interactions allows the derivation of thermodynamic models where temperature and density serve as independent variables.^1–3^

For a system composed of different pure substances, the second virial coefficient depends on the composition and can be written as^21^

$$B_{\mathrm{mix}}(T)=\sum_{ij}x_{i}x_{j}B_{ij}(T)$$ 5

where *B_ij_* (*T*) represents the second virial coefficient associated with the interaction between the components *i* and *j*, and *x*_*i*_ and *x*_*j*_ are the mole fractions of each component of the mixture satisfying *x_i_* + *x_j_* = 1.

It is important to stress that the present work does not evaluate the full virial coefficient of the mixture *B*_mix_(*T*), but rather specific contributions corresponding to pure systems or cross-interactions, depending on the available potential energy surface (PES). Throughout this work, we omit the explicit subscripts in *B*(*T*), but it is implicitly understood that, unless otherwise stated, we are referring to the cross virial coefficient.

According to eq 5, the second virial coefficient of a binary mixture N_2_ + H_2_ requires three contributions:

$$B_{N_{2}+H_{2}}=x_{N_{2}}^{2}B_{N_{2}\cdot \cdot \cdot N_{2}}+2x_{N_{2}}x_{H_{2}}B_{N_{2}\cdot \cdot \cdot H_{2}}+x_{H_{2}}^{2}B_{H_{2}\cdot \cdot \cdot H_{2}}$$ 6

However, with the PES adopted here, it is possible to calculate only the cross contribution, *B*_N_2_···H_2__, which describes the interaction between N_2_ and H_2_. The remaining terms *B*_N_2_···N_2__ and *B*_H_2_···H_2__ would require distinct PESs for the corresponding homonuclear interactions. Thus, in this case, our results refer specifically to the cross virial coefficient and not to the virial coefficient of a mixture.

For the NH dimer, the system corresponds to a single pure substance. Therefore, the second virial coefficient directly coincides with the interaction contribution:

$$B_{\mathrm{NH}+\mathrm{NH}}=B_{\mathrm{NH}\cdot \cdot \cdot \mathrm{NH}}$$ 7

#### Second Virial Coefficient

We focus on the second term in eq 3, representing the first-order correction to the ideal gas law. This linear approximation captures the behavior of real gases at low pressures. The second virial coefficient, *B*(*T*), is given by^1,3,22^

$$B(T)=-\frac{1}{2}\int \int d^{3}r_{1}d^{3}r_{2}f_{12}$$ 8

where *f*_12_ is the Mayer function, *u*_12_ is the pair potential depending on intermolecular distance and orientation, β = 1/*k*_B_ *T*, and *k*_B_ is the Boltzmann constant. This classical formulation is highly advantageous for rapid estimates and remains accurate in high-temperature regimes, where quantum corrections are typically less than 1%.^23^ Although quantum corrections to the second virial coefficient have been computed for simpler one-dimensional systems,^24,25^ their inclusion is beyond the scope of the present work due to the complexity and strong anisotropy of the interaction potential.

In the diatom–diatom Jacobi coordinates, the classical cross-second virial coefficient can be expressed as^26^

$$B(T)=-\frac{N_{A}}{4}\int_{0}^{2\pi }d\phi \int_{0}^{\pi }\sin\theta_{2}d\theta_{2}\int_{0}^{\pi }\sin\;\theta_{1}d\theta_{1}\times \int_{0}^{\infty }r_{12}^{2}dr_{12}[1-\exp(-\beta u(r_{12},\theta_{1},\theta_{2},\phi ))]$$ 9

where *N*_A_ is Avogadro’s number and *u*(*r*_12_, θ_1_, θ_2_, ϕ) is an anisotropic interaction potential depending on intermolecular distance and orientation, referred to the corresponding dissociation channel. While the radial integration ideally extends to infinity, a critical issue arises at very short distances. For specific angular orientations, the potential develops a deep attractive well that diverges as *r*_12_ → 0, as illustrated in Figure 2. Consequently, the Mayer function *f*_12_ diverges over a finite range of distances, leading to divergences in *B*(*T*). This effect is not a numerical artifact, but reflects a physical phenomenon in which the interactions reach a state of extreme stability, specifically the formation of a stable chemical bond (e.g., H–N=N–H in the short-range region), which falls outside the virial coefficient approximation. For example, the *cis*-structure H–N=N–H, corresponding to the geometry θ_1_ = 2π/3, θ_2_ = π/3, and ϕ = 0, with an energy of approximately 0.2*E*_h_ below the NH­(X^3^Σ_u_^–^) + NH­(X^3^Σ_u_^–^) dissociation channel. Other configurations also exhibit an attractive well on the order of 10^–1^ *E*_h_, as shown in Figures S3 and S4 of the SM. This high energy scale is characteristic of chemical processes, not a gas-phase like interaction, typically several orders of magnitude smaller (around 10^–4^ Hartree).

To address the divergences mentioned in the previous paragraph, we introduce a physically motivated, orientation-dependent cutoff, σ­(θ_1_, θ_2_, ϕ), which separates two integration regions in eq 9:

$$B(T)=-2\pi N_{A}\left[\int_{0}^{\sigma }\langle f(r_{12})\rangle_{\Omega }r_{12}^{2}dr_{12}+\int_{\sigma }^{\infty }\langle f(r_{12})\rangle_{\Omega }r_{12}^{2}dr_{12}\right]$$ 10

where the angularly averaged Mayer function is defined as

$$\langle f(r_{12})\rangle_{\Omega }=\frac{\int_{0}^{2\pi }\int_{0}^{\pi }\int_{0}^{\pi }f(r_{12},\theta_{1},\theta_{2},\phi )\sin\theta_{1}\sin\theta_{2}d\theta_{1}d\theta_{2}d\phi }{\int_{0}^{2\pi }\int_{0}^{\pi }\int_{0}^{\pi }\sin\theta_{1}\sin\theta_{2}d\theta_{1}d\theta_{2}d\phi }$$ 11

For 0 ≤ *r*_12_ < σ, we employ an approximated potential that captures the strong short-range attraction while keeping the integral finite. For *r*_12_ ≥ σ, the full anisotropic potential is used, which is well-behaved and convergent. This approach differs fundamentally from isotropic models (e.g., Lennard-Jones), since σ depends explicitly on angular orientation. The specific determination of σ and the construction of the approximated potential are presented in the Results section.

### Thermodynamic Properties

#### Residual Functions

The thermodynamic state of a system can be described using different sets of independent variables, such as (*T*, *V*, *n*) or (*T*, *p*, *n*). A general property *X* can then be written in terms of these variables. The residual function represents the deviation of a real gas property from its ideal gas counterpart:

$$X^{\mathrm{res}}(T,V,n)=X(T,V,n)-X^{\mathrm{pg}}(T,V,n)$$ 12

$$X^{\mathrm{res}}(T,p,n)=X(T,p,n)-X^{\mathrm{pg}}(T,p,n)$$ 13

The two representations are related via:

$$X^{\mathrm{res}}(T,V,n)=X^{\mathrm{res}}(T,p,n)+\int_{p_{r}}^{p}{\left(\frac{\partial X^{\mathrm{pg}}}{\partial p}\right)}_{T,n}dp$$ 14

where *p*_r_ = ρ_n_ *RT* is the reference pressure. From the virial expansion, residual properties of pure gases and mixtures can be calculated. Their evaluation requires derivatives of the compressibility factor *Z* and of *B*(*T*), as given by eq 9. Expressions for residual functions of the thermodynamic properties can be found elsewhere.^22,27,28^

The compressibility factor can be expressed in terms of molar volumes as^19^

$$Z=\frac{V_{m}}{V_{m}^{\mathrm{pg}}}$$ 15

with *V*_m_ = *V*_m_^res^ + *V*_m_^pg^. In the low-pressure limit:

$$\lim_{p\to 0}V_{m}^{\mathrm{res}}=B(T)$$ 16

The condition *B*(*T*) = 0 signifies a balance between repulsive and attractive interactions, where real gases mimic ideal gas behavior. This occurs at the Boyle temperature *T*_B_, defined by *Z* = 1. For *Z* > 1, repulsive interactions dominate; for *Z* < 1, attractive interactions prevail.

Departures from ideality are also evident in the heat capacities. For real gases, the relation *C*_p_ – *C*_v_ = *R* does not hold. The residual heat capacities are given by^1^

$$C_{v}^{\mathrm{res}}=-R\left(\frac{2T}{V\sim }\frac{dB}{dT}+\frac{T^{2}}{V\sim }\frac{d^{2}B}{dT^{2}}\right)$$ 17

$$C_{p}^{\mathrm{res}}=-\frac{{RT}^{2}}{V\sim }\frac{d^{2}B}{{dT}^{2}}+\frac{R}{V\sim^{2}}{\left(B-T\frac{dB}{dT}\right)}^{2}$$ 18

#### Inversion Temperature

Consider a throttling process in which a gas passes through a porous plug inside an insulated tube. According to the first law of thermodynamics, in the absence of heat exchange, any change in internal energy corresponds to the work performed. Experiments indicate that temperature changes occur during throttling due to pressure variations, a phenomenon known as the Joule–Thomson effect. Notably, this process occurs at constant enthalpy (Δ*H* = 0), a behavior not predicted by the ideal gas law.

The Joule–Thomson coefficient is defined as^29,30^

$$\mu_{J}={\left(\frac{\partial T}{\partial p}\right)}_{h}=-\frac{1}{c_{p}}{\left(\frac{\partial h}{\partial T}\right)}_{p}$$ 19

Here, *c*_p_ denotes the isobaric specific heat and *h* the specific enthalpy. In real gases, μ_J_ ≠ 0 because enthalpy and internal energy depend on *T*, *p*, and *V*, providing a direct measure of the deviation from ideal behavior. At low pressures:

$$\lim_{p\to 0}\;\mu_{J}=\frac{1}{c_{p}^{0}}\left(T\frac{dB}{dT}-B\right)$$ 20

The condition for minimum enthalpy yields the inversion temperature *T*_*i*_:

$$B(T_{i})=T_{i}{\left(\frac{dB}{dT}\right)}_{T=T_{i}}$$ 21

At *T* = *T_i_*, the Joule–Thomson coefficient changes sign. For ideal gases, μ_J_ = 0 at all temperatures.

## Results and Discussion

Within the rigid-body approximation, the NH and N_2_···H_2_ dimers are treated with fixed interatomic distances: *R*_NH_ = 2.350*a*_0_ for NH, and *R*_NN_ = 2.074*a*_0_ and *R*_HH_ = 1.401*a*_0_ for N_2_···H_2_. This simplification reduces the dimensionality of the system from six to four, thereby enhancing computational efficiency. To maintain consistency in the numerical integration over the interval [0, *∞*), a variable transformation was applied to transform it to [0, 1]. Despite this, the complexity of the potential continues to pose convergence challenges in the evaluation of the Mayer function.

The radial integration of the virial coefficient is performed by splitting the range into two distinct regions, as previously discussed in eq 10. For the short-range part of the integral, our approach differs on the basis of the nature of the potential. For purely repulsive configurations, we model the interactions for *r* < σ using a rigid-sphere approximation, as the contribution from this region to the integral is negligible. For attractive configurations, where the short-range potential may lead to divergences, we introduce a modified potential for *r* < σ that preserves the strong short-range attraction while ensuring the integral remains finite. The long-range portion of the integral (*r* ≥ σ) is truncated at a finite upper limit of 40 *a*_0_, since the rapid decay of the potential beyond this distance ensures convergence of the integral, as verified using the Romberg method.^31^ For the numerical integration, a 10-point Gaussian quadrature was employed for the angular variables, whereas the Romberg method was applied to the radial integration. The number of angular and radial points was incrementally increased until the results met the established convergence criteria, ensuring precision and reliability in the calculations.

For the Gaussian quadrature,^31^ convergence was assessed by the stability of the integral value upon increasing the number of quadrature points. Specifically, iterations continued until the absolute difference between integrals computed with *n* and 2*n* points satisfied $\frac{\left|I_{n}-I_{2n}\right|}{\left|I_{n}\right|}<10^{-6}$ , ensuring sufficient precision in the angular integration. For the Romberg method,^31^ convergence was assessed through the extrapolation process: iterations were stopped when the relative difference between two consecutive extrapolated values, *R*_*k*,*k*_ and *R*_*k–*1,*k–*1_, satisfied the condition $\frac{\left|R_{k,k}-R_{k-1,k-1}\right|}{\left|R_{k,k}\right|}<10^{-8}$ . These criteria ensured precise and reliable results across the domain of integration. The values *R*_*i*,*j*_ in Romberg integration represent the integral approximations at different refinement and extrapolation levels, calculated based on the trapezoidal rule with progressively smaller subdivisions of the integration interval.

The van der Waals (vdW) interaction, as described by Halgren,^32^ constitutes a fundamental component in modeling nonbonded interactions within molecular mechanics force fields. In this framework, the potential energy surface of dimers exhibits multiple vdW minima, each associated with a characteristic energy scale that defines the strength of the intermolecular interactions. Specifically, the DMBE-PES^11^ it was observed that the NH dimer has a van der Waals (vdW) minimum 768 cm^–1^ below the dissociation channel NH­(^3^Σ^–^) + NH­(^3^Σ^–^) when θ_1_ = θ_2_ = 0. This corresponds to a distance of the order of 12.20 *a*_0_. These values are consistent with the CASPT2 results reported in reference,^33^ namely 718 cm^–1^ (3.27 × 10^–3^ *E*_h_) and 12.26 *a*_0_, respectively. The channel N_2_(X^1^Σ_g_^+^) + H_2_(X^1^Σ_g_^+^) has two vdW minima. The first minimum occurs when θ_1_ = θ_2_ = 0 and in this case *r*_min_ = 14.11*a*_0_ with a well depth of 88 cm^–1^ (4.01 × 10^–4^ *E*_h_). The second occurs when θ_1_ = θ_2_ = π/2 with *r*_min_ = 12.24*a*_0_ and a well depth of 22 cm^–1^ (1.00 × 10^–4^ *E*_h_).

These vdW well depths are on the order of 10^–4^ to 10^–3^ Hartrees. This energy scale is different from the energy associated with chemical bonds, such as the N_2_ potential well depth, which is on the order of 10^–1^ Hartree. This disparity highlights that the virial expansion applies to vdW interactions, but not to the formation of bound states with energies comparable to chemical bonds, necessitating the careful selection of the cutoff distance.

Hence, numerical integrations were performed for various values of σ between 4.5 and 6.0 *a*_0_. This result is consistent with previous molecular dynamics studies using the same PES.^14^ Different approaches have been used in the literature to deal with this cutoff distance. A hard-sphere model, with a fixed diameter, is usually employed in the short-range limit of the interaction potential.^5,6,8,34^

However, the upper limit of integration for the radial coordinate (*r*) was defined as 40*a*_0_. This value is not a fixed boundary but represents a practical limit; at this distance, the interactions between the dimers become negligible, that is, the expression lim_*r*→*∞*_ *u*(*r*) is the asymptotic limit of the corresponding dissociative channel. For the angular variables θ_1_ and θ_2_, the integration takes place between 0 and π. While for the variable ϕ the integration is performed between 0 and 2π. Considering there is more data available for the N_2_···H_2_ system, the calculation procedure will first be tested upon it. Subsequently, such a procedure will be used for NH···NH.

### N_2_···H_2_

The results of *B*(*T*) for N_2_···H_2_ have approximate values compared to the corresponding reported in refs 21 and 35 in the temperature range from 36 and 350 K. Figure 3 illustrates the behavior of *B*(*T*) as a function of absolute temperature. The lines represent the second virial coefficient value *B* ^cal^ calculated from the DMBE-PES. The red, blue, and black lines are related to the values of *B*(*T*) for σ covering the interval 5.0–6.0*a*_0_. It is of interest (see later) to report also the average value $\overset{®}{B(T)}$ also for this range of σ, represented with a green line. The dots represent the experimental values *B* ^exp^. From Figure 3, for temperatures below 100 K, all calculated second virial coefficients are in good agreement with experimental data, and differences due to the selection of σ are negligible. In turn, for temperatures between 100 and 300 K, the values of *B* ^cal^ are very sensitive to the selection of σ. Table S1 in the Supporting Information (SM) summarizes *B*(*T*) for the temperature range here studied and 5.4 ≤ σ/*a*_0_ ≤ 5.8. For completeness, Table S2 in the SM collects the calculated values for temperatures 290 ≤ *T*/K ≤ 350 and 5.0 ≤ σ/*a*_0_ ≤ 5.4. In Tables S1 and S2, the experimental data from ref 35. can be used to validate the methodology followed here. In turn, the results by Tat and Deiters^26^ provide support for our methodology as it uses an ab initio-based intermolecular potential energy surface of N_2_···H_2_ dimer to calculate the second virial coefficient of the interaction of N_2_ and H_2_ pairs. For comparison, the calculated values of the second virial coefficient *B*(*T*) from ref 21. are also presented in Tables S1 and S2 in the SM.

**Figure 3:**
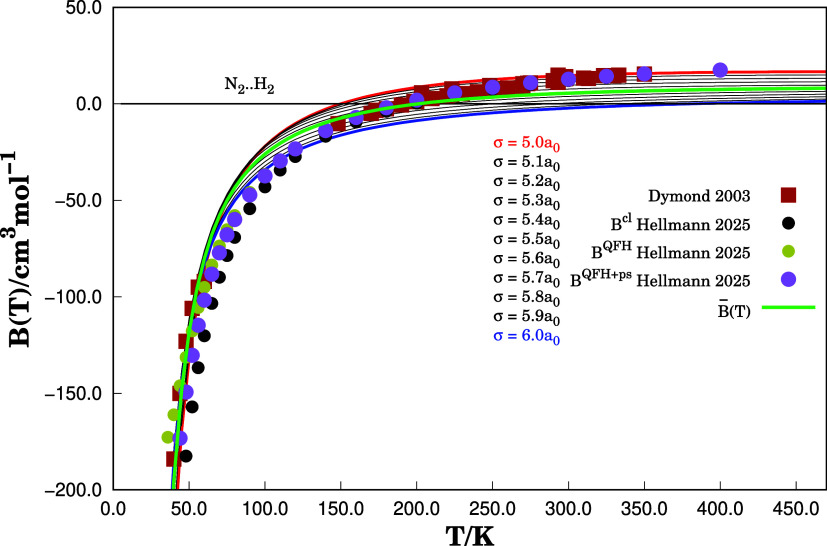
Interaction second virial coefficient *B*(*T*) as a function of temperature for the N_2_···H_2_ system. Solid lines represent calculated values from eq 9 using the DMBE-PES for different values of σ. Filled squares (■) correspond to experimental data from ref 35, while filled circles (●) denote classical and quantum-corrected results from ref 23. The green curve represents the average *B*(*T*) obtained in this work.

The inspection of Figures S3, S4, and Table S1 in the SM shows that *B* ^cal^ at 40 and 50 K temperatures present relative errors of 0.59 and 0.69%, respectively, while the theoretical work of ref 21. presented relative errors of 23.83 and 12.79% compared to *B* ^exp^(*T*).^35^ For temperatures between 130 and 350 K, the second virial coefficient was calculated with errors less than 15 cm^3^ mol^–1^. From the graphs presented in Figure S4, the best value of σ to reproduce the experimental value of *B*(*T*) changes with temperature. Thus, fixing the same value of σ for all temperatures is not a good choice.

For a deeper analysis, a correlation between the calculated and the experimental values of *B*, using Pearson’s correlation coefficient, was carried out. Figure S1 displays a scatter plot for *B* ^exp^ and *B* ^cal^. In general, the second virial coefficients calculated are strongly correlated to the experimental data: a correlation coefficient close to +1. However, for low temperatures and negative *B*, the selected value of σ largely influences *B* ^cal^. Therefore, not all values of σ are recommended for calculating the second virial coefficient. For the values of *B*(*T*) obtained from eq 9, with σ = 5.5*a*_0_, we obtain a correlation coefficient of 0.996, which indicates a very strong positive correlation when compared to the experimental data. For further comparisons with the available data, root-mean-square-deviation (RMSD) was also calculated and displayed in Figure S2 in the SM.

We cannot define the optimal value for σ from these results. Instead, it is interesting to determine the average value of *B*(*T*) for each temperature value, taking into account the values of σ between 5.0 *a*_0_ and 6.0 *a*_0_. From calculations in the temperature range between 45 and 95 K, the changes of the second virial coefficient *B*(*T*), calculated with several values of σ, are less than 30% relative to the corresponding average. Compared to the experimental values, the best results for the second virial coefficient are obtained for 5.4 < σ/*a*_0_ < 5.8. However, this is not generally applied to the entire temperature range here studied: e.g., for σ = 5.4*a*_0_ *B*(*T*) is obtained with a relative error larger than 40% at *T* = 350 K, in turn, at *T*= 44 K the corresponding error is around 3%. In summary, a detailed analysis of the calculation error of the second virial coefficient must be taken into account since the RMSD is not sufficient for a complete conclusion regarding the integration of the eq 9. In this sense, it is verified that at temperatures close to 250 K a second virial coefficient is reproduced with an error smaller than 5 cm^3^ mol^–1^ when we fix the values σ = 5.3*a*_0_ or σ = 5.4*a*_0_. This corresponds to a relative error of less than 20% in the *B*(*T*) calculation. On the other hand, when the system temperature is around 300 K, a better representation of *B*(*T*) is observed if σ = 5.1*a*_0_ since the deviations are around 1 cm^3^ mol^–1^. For *T* = 350 K and σ = 5.0*a*_0_ we found absolute and relative errors of 0.35 cm^3^ mol^–1^ and 2.3% respectively.

We recommend a fitted function for the cross second virial coefficient of N_2_···H_2_ based on the calculated *B*(*T*) values, averaged over the variations of the intermolecular distance, σ. The fit is given by *B*_fit_(*T*) = 35.2782 – 7.59661 × 10^3^/*T* + 2.23636 × 10^5^/*T* ^2^ – 1.04854 × 10^7^/*T* ^3^, with *B*_fit_(*T*) in cm^3^ mol^–1^ and *T* in Kelvin. Deviations relative to the reference values are provided in Figures S5 and S6 of the Appendix for completeness.

### NH···NH

The interaction between NH diatoms can be studied based on the potential energy surface used in this work. To our knowledge, no global PES has been reported for the triplet and quintet electronic states. In turn, a previous study presented ab initio PESs for these three electronic states, for fixed configurations of the NH radical.^33^ The three PES adiabatically approach in the long-range region, which is accurately described with the singlet PES. Hence, one expects to get a quantitative representation of the thermodynamic properties from the singlet PES. Thus, the present work considers only the electronic singlet state of N_2_H_2_.

As it is a pure substance, we do not need to be concerned about partial fractions; this way, it is possible to calculate some contributions to thermodynamic properties. A preliminary observation must be made: the results obtained for the second virial coefficient only refer to the contribution of interactions between pairs. In other words, in this work, we do not calculate the contributions arising from collisions, which may be reasonably significant in obtaining the thermodynamic properties, as we will see later.

After a study of the topological characteristics of PES, we choose 4.7 ≤ σ/*a*_0_ ≤ 4.8. In this case, the differences relative to the averaged value are less than 30% for temperatures above 800 K. That is, a change in the parameter σ in the integral of eq 9 does not significantly affect the value of *B*(*T*). In turn, for temperatures below 800 K, *B* ^cal^ strongly depends upon the selected value of σ. Nonetheless, the absence of experimental data precludes a rigorous quantitative comparison of *B*(*T*). Instead, the two-interacting dipole model can be used as a first approximation for the NH dimer. Analytical models for representing potential energy surfaces, such as Stockmayer’s, suggest that in polar systems, the virial’s second coefficient depends on the diatom’s permanent dipole moment.^36^

For instance, molecules with permanent dipole moments interact via dipole–dipole forces, significantly affecting the second virial coefficient, particularly in polar gases. A larger dipole moment enhances the attractive intermolecular forces, often resulting in a more negative *B*(*T*).

In this sense, we take the dimers of CO, HCl, NH_3_, and H_2_O as a reference to make qualitative comparisons. The literature provides us with several experimental and theoretical data on these dimers. Thus, to calibrate the σ parameter in the calculation of the integral, we use as an argument the fact that the dipole moments obey the following relationship: μ_CO_ < μ_HCl_ < μ_NH_3__ < μ_NH_ < μ_H_2_O_.^11,37–40^ We emphasize that all interactions of the multipolar nature of the NH···NH system are included in the PES. The philosophy of the DMBE method is to expand the interatomic potential into a short-range and a long-range. On the other hand, as set out above, it is expected that the behavior of the thermodynamic properties of NH and other polar dimers present in this work is comparable. Thus, the values of *B*(*T*) are calculated for values of σ between 4.7 and 4.8 *a*_0_ as shown in Figure 4. With these values of σ, we will construct a confidence interval for *B*(*T*) as a function of *T*. However, in the calculations of thermodynamic properties, we will use the value σ = 4.7 *a*_0_.

**Figure 4:**
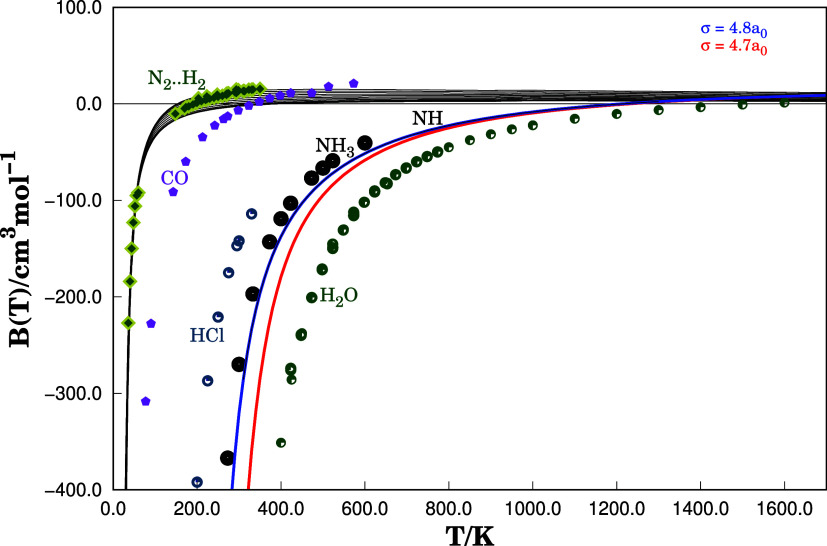
Cross second virial coefficient for different substances with comparable molar mass values. It is observed that the multipolar character interferes with the behavior of *B*(*T*) for these systems, shifting the Boyle temperature value from left to right according to the direction of growth of the permanent dipole moment.

We can determine some thermodynamic properties once we have defined a consistent way to calculate *B*(*T*). First, we can estimate the Boyle temperature for this system. In the case of the NH···NH structure, the cancellation between attractive and repulsive forces, that is to say, *T*_B_, occurs within a specific temperature range 1210 < *T*_B_/K < 1230. The Table 1 collects the values of *T*_B_ for CO, HCl, NH_3_, and H_2_O dimers reported in Literature. Notably, the values of *T*_B_ reported for these systems follow the same order as the dipole moments, as expected. Figure 4 displays *B*(*T*) for selected systems to extend the comparison with other dipole–dipole-dominated dimers. The Boyle temperature is shifted to higher temperatures as the dipole of the monomer increases. The N_2_···H_2_ interaction, which presents a case of the zero dipole moment limit, was also included in Figure 4.

**Table 1:** Temperatures of Boyle (*T*_B_), of Inversion (*T_i_*), and the *T_i_*/*T*_B_ for Different Dimers

system	*T*_B_/K	*T_i_*/K	*T_i_*/*T*_B_	ref
CO···CO	347	653	1.884	^21^
	337	648	1.921	^41^
	342	674	1.969	^41^
	343			^42^
	345			^43^
HCl···HCl		1653		^44^
NH_3_···NH_3_	1034			^42^
		1901		^44^
NH···NH	1230^a^	2045	1.662	this work
	1210^b^	2020	1.669	this work
H_2_O···H_2_O	1512			^45^
	1599			^42^
		2538		^44^

a σ = 4.7.

b σ = 4.8.

For temperatures below *T*_B_, the virial correction should be negative because the volume occupied is smaller than that predicted by the theory of perfect gases. This characterizes an attractive effect between diatoms, where the average distance between them decreases with decreasing temperature. That is, in the limit where the pressure tends to zero, the residual volume is negative below the Boyle temperature. For temperatures above *T*_B_, the effect is repulsive. In this case, the residual volume is positive when the dimer is subjected to a temperature above *T*_B_.

From the second virial coefficients for a given temperature range, the compressibility factor *Z* for the NH dimer can be calculated directly from eqs 3 and 4. The dimer compressibility factor shows a significant dependence on the system pressure. From the truncation of the expansion in eqs 3 and 4, it is expected that *Z* linearly depends upon the pressure as shown in Figure 5. Furthermore, the temperature to which the dimer is subjected considerably affects the behavior of *Z*. Comparing Figure 5 with Table 1, it is observed that at Boyle temperature *Z* → 1, a fact that is characterized in the graph in which the slope of *Z*, as a function of *p* or ρ, vanishes. This indicates the temperature and pressure range over which the system behaves as an ideal gas.

**Figure 5:**
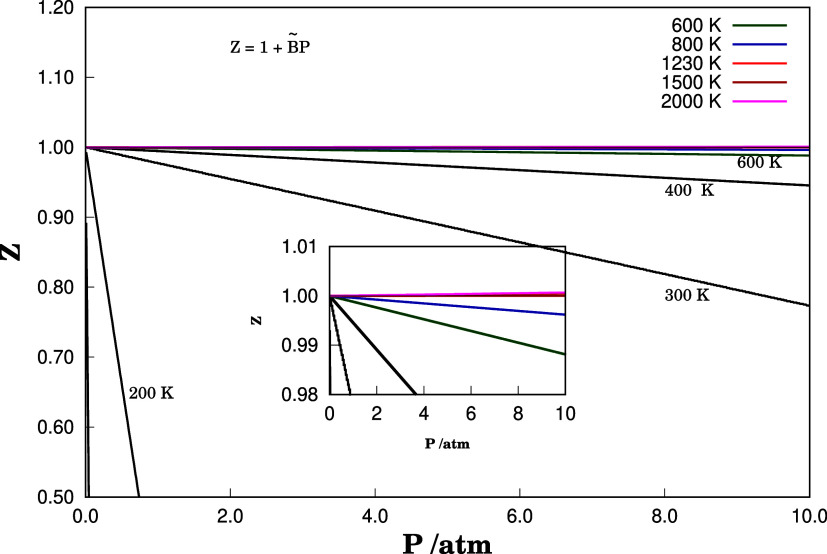
Compressibility factor *Z* was calculated for the NH···NH dimer at different temperatures. In red is the Boyle temperature of 1230 K. Larger deviations from ideality are observed for low temperatures and high pressures.

For temperatures close to 200 K, the system tends to have a more repulsive behavior between NH diatoms. This contrasts with the perfect gas model since it does not take into account any type of interaction between the gas constituents. Qualitatively, it is observed that when the NH dimer is confined to temperatures below 200 K, for example, the perfect gas model should not be used to describe the system.

In thermodynamics, the difference between the heat capacities at constant pressure (*C*_p_) and constant volume (*C*_v_) is a significant characteristic of real gases that deviates from the behavior of ideal gases. This difference is linked to how the internal energy and the work done by the gas during expansion respond to changes in temperature and pressure.

For ideal gases, the difference *C*_p_ – *C*_v_ is equal to the universal gas constant, *R* = 8.315 J K^–1^ mol^–1^, i.e., *C*_p_ – *C*_v_ = *R*. However, for real gases, this relationship is altered due to intermolecular interactions and the finite volume of gas molecules, resulting in more complex behavior. The forces of attraction or repulsion between molecules influence the internal energy of the gas, thus changing the amount of heat needed for temperature variations under constant pressure and constant volume conditions. Consequently, real gases do not behave in a perfectly expansive manner; their compressibility varies with pressure and temperature, affecting the work performed during expansion. Eqs 12, 17, and 18 are used to determine the difference *C*_p_ – *C*_v_, and the results are plotted in Figure 6. The corresponding pressures are indicated on the left side of each curve, and the ideal gas limit is also shown.

**Figure 6:**
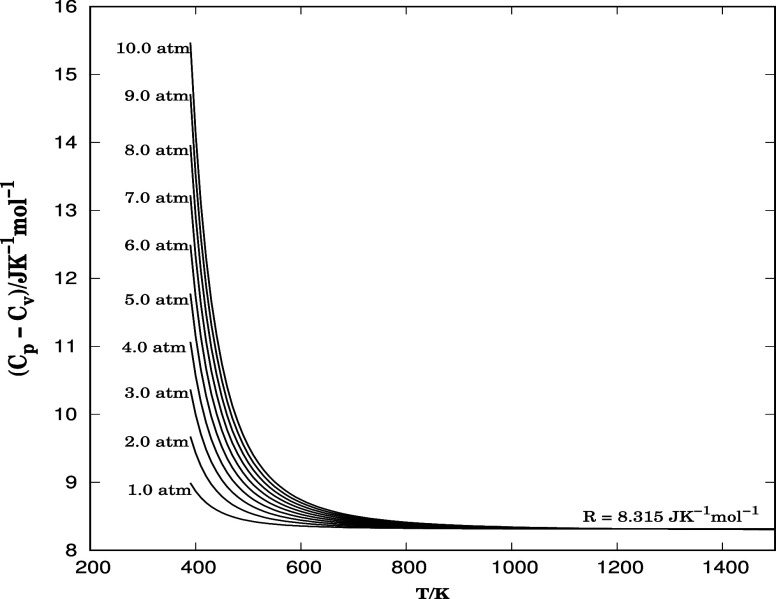
*C*_p_ – *C*_v_ in terms of temperature and pressure. Values of *C*_p_ and *C*_v_ obtained from the residual function with the compressibility factor truncated are given in J K^–1^ mol^–1^. The corresponding pressure is given in atm on the left side of each curve atm.

The eq 20 vanishes when the condition of eq 21 is fulfilled. This means that at inversion temperature, *T_i_*, the solution of eq 21 is given by a tangent line that passes through the origin as shown in Figure 7. From eq 19 and Figure 7, it is possible to theoretically observe the Joule-Thomson effect in an isoenthalpic strangulation process. Negative values of μ_J_ mean heating in the gas when the pressure decreases; in contrast, for positive values of μ_J_, we have a cooling of the gas. When *T* = *T_i_*, μ_J_ = 0, then the values of *T_i_* are obtained by numerically solving eq 20. The obtained values are reported in Table 1. Also, the corresponding *T_i_* s for polar fluids were reported in the Table 1. We emphasize that if the system is at the inversion temperature *T_i_* and subjected to low pressures, the enthalpy of the gas does not depend on the pressure. However, this does not mean that the internal energy is also independent of pressure, since the condition *B* ≠ 0 is valid, the gas will deviate from a perfect gas behavior. Finally, from the van der Waals equation of state, the Boyle and the inversion temperatures are related as follows *T_i_* = 2*T*_B_.^46^ However, what is generally observed is an approximate relationship *T_i_* ≤ 2*T*_B_. The latter is also confirmed in the calculations reported here.

**Figure 7:**
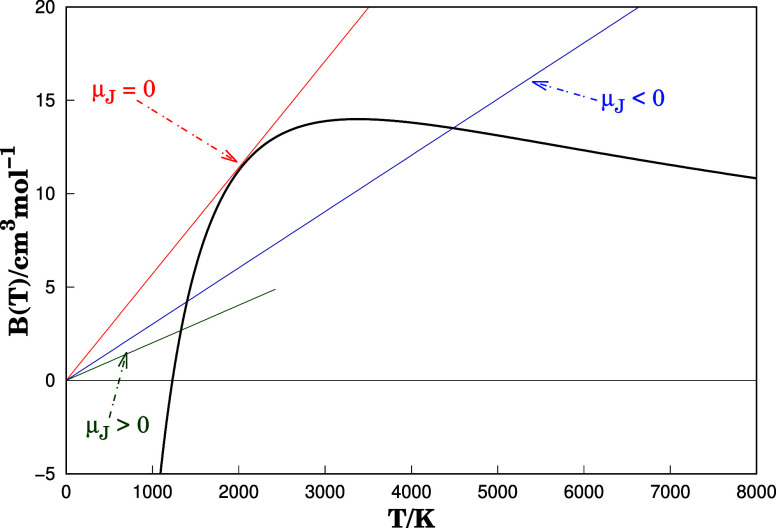
Second virial coefficient *B*(*T*) against temperature for NH···NH. The graph shows the sign of the Joule–Thomson coefficient. The inversion temperature is obtained when μ_J_ is zero. Our calculation shows that *T*_B_ = 1230 K and *T_i_* = 2045 K. The methods converge when the second virial coefficient calculated is less than 10^–4^ cm^3^ mol^–1^.

## Conclusions

This work calculated the second virial coefficients for the diatomic pairs NH···NH and N_2_···H_2_ using the cluster integral approach. The calculation methodology was validated by reproducing previously reported results for N_2_···H_2_, and subsequently applied to the NH···NH dimer. A critical aspect of this calculation is the careful selection of the smallest intermolecular distance, σ, which represents the minimum approach distance between the interacting diatoms. The choice of σ must balance numerical accuracy with the physical characteristics of the interaction potential, as well as consistency with available experimental data for similar systems. Notably, the results suggest that σ may exhibit some orientation dependence, as observed for the systems studied. The results further demonstrate a pronounced dependence of *B*(*T*) on the permanent dipole moment of the systems, particularly for polar molecules. This trend is observed in the case of the NH···NH dimer, where the theoretical *B*(*T*) curve lies between those of NH_3_ and H_2_O, consistent with their respective dipole moments. The main source of discrepancies between our theoretical predictions and the experimental data arises from the use of a purely classical formulation of the cross second virial coefficient, without quantum corrections.

## Supplementary Material



## Notes

The Supporting Information contains additional tables, statistical analysis, and validation of the calculated coefficients (PDF)
