# Supplementary material for: Second Virial Coefficients for N2···H2 and NH···NH
Source: J Phys Chem A. 2026 Jan 2;130(2):387–96. doi: 10.1021/acs.jpca.5c04624 (PMC12814513; doi:10.1021/acs.jpca.5c04624)
Supplement: Supplementary file 1 [file jp5c04624_si_001.pdf]

Second virial coefficients for $N_2 \cdots H_2$ and $NH \cdots NH$

Supplementary Material

Marcos D. S. Alves Maikel Y. Ballester*

Departamento de Física,

Universidade Federal de Juiz de Fora,

MG, Brazil

(Dated: November 3, 2025)

* maikel.ballester@ufjf.br

This supplementary material (SM) summarizes B(T) for the temperature range here studied and $5.4 \leq \sigma/a_0 \leq 5.8$. For completeness, Table S2 collects the calculated values for temperatures $290 \leq T/K \leq 350$ and $5.0 \leq \sigma/a_0 \leq 5.4$. In tables S1 and S2, the experimental data from Ref. 1 can be used to validate the methodology followed here. In turn, the results by Tat and Deiters [4] provide support for our methodology as it uses an ab initio-based intermolecular potential energy surface of $N_2 \cdots H_2$ dimer to calculate the second virial coefficient of the interaction of N_2 and H_2 pairs. For comparison, the calculated values of the second virial coefficient B(T) from Ref. 2 are also presented in Tables S1 and S2.

S1. POTENTIAL ENERGY SURFACE

The Double many-body expansion (DMBE) potential energy surface (PES) employed in this work was previously developed by Poveda, Biczysko, and Varandas [3]. The DMBE-PES for the ground electronic state of N_2H_2 dissociate adiabatically to channels:

$$\mathrm{NH}(X^3\Sigma_u^-) + \mathrm{NH}(X^3\Sigma_u^-) \tag{1}$$

$$\mathrm{HN_2}(X^2A') + \mathrm{H}(^2S) \tag{2}$$

$$\mathrm{N_2}(X^1\Sigma_g^+) + \mathrm{H_2}(X^1\Sigma_g^+) \tag{3}$$

$$\mathrm{N}(^2D) + \mathrm{NH_2}(^2A'') \tag{4}$$

$$\mathrm{N}(^2\mathrm{S}) + \mathrm{NH_2}(^4A'') \tag{5}$$

$$\tag{6}$$

using switching functions schemes, and many-body terms accurately reproducing each di and triatomic fragment, see Ref. [3] for more details.

To calculate the virial coefficient in Equation (10) of the manuscript, it is necessary to adapt the potential energy surface. That is,

$$V_{N_2H_2}(\vec{R}) \longrightarrow u(r_{12}, \theta_1, \theta_2, \phi),$$

where the original potential energy surface (SEP) was constructed according to the inter-particle coordinate scheme:

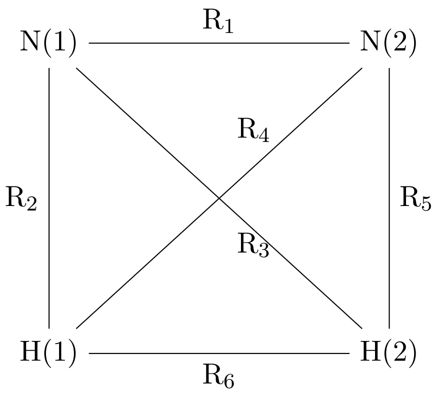

FIG. S1. Interparticle coordinate system

The six distances are defined as:

$R_1 = R_{N_{(1)}N_{(2)}}$

$R_2 = R_{N_{(1)}H_{(1)}}$

$R_3 = R_{N_{(1)}H_{(2)}}$

$R_4 = R_{N_{(2)}H_{(1)}}$

$R_5 = R_{N_{(2)}H_{(2)}}$

$R_6 = R_{H_{(1)}H_{(2)}}$

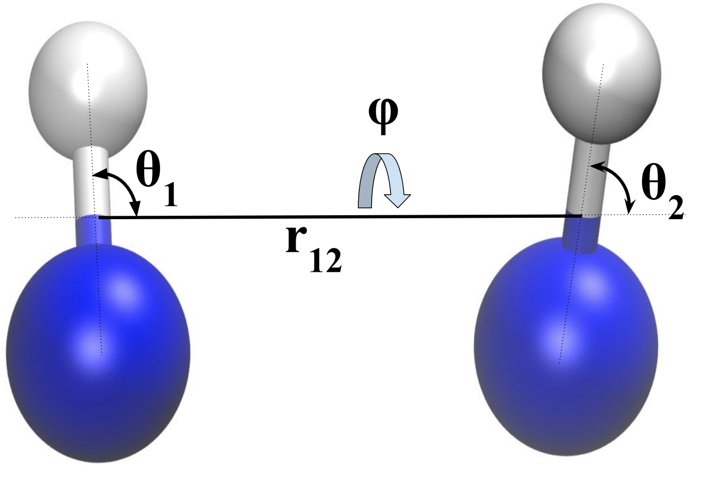

FIG. S2. Jacobi coordinates for the diatom–diatom system.

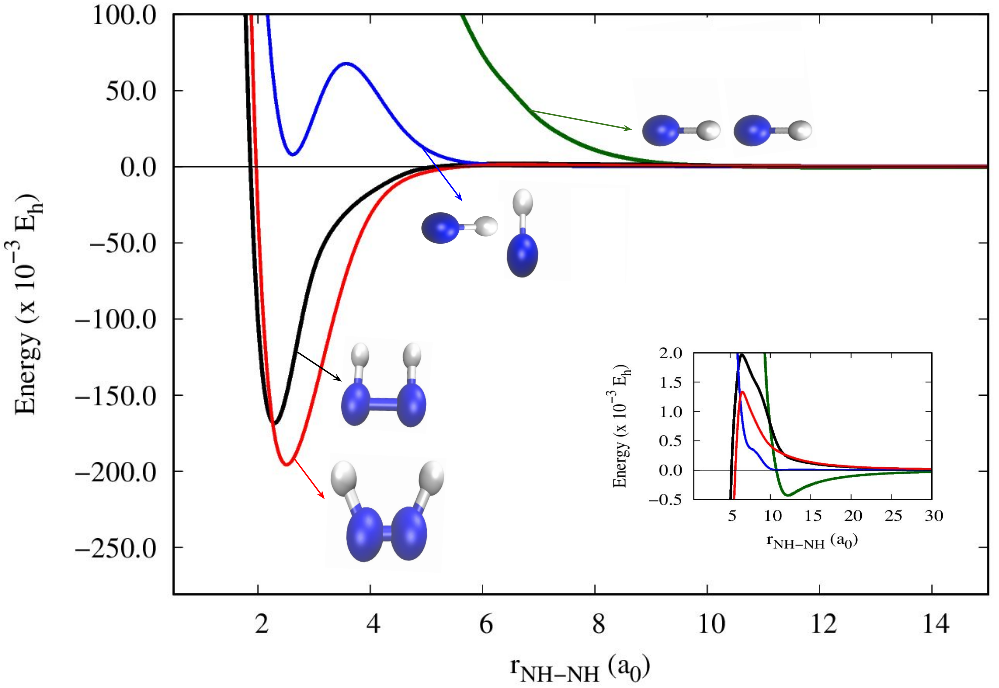

FIG. S3. **Methodological justification for σ: Delimitation of energy scales in the PES.** The graph (main panel) illustrates the energy interaction as a function of the intermolecular distance between two NH diatomic molecules for four different orientations, contrasting van der Waals interactions (inset panel) with chemical bond formation. Black and red curves correspond to the geometry ($\theta_1 = \pi/2$, $\theta_2 = \pi/2$, $\phi = 0$) and ($\theta_1 = 2\pi/3$, $\theta_2 = \pi/3$, $\phi = 0$) respectively, which represent the cis H-N=N-H structure, which shows a deep energy well, characteristic of a stable chemical bond. Green curve corresponds to the geometry ($\theta_1 = 0$, $\theta_2 = 0$, $\phi = 0$) which exhibits a vdW minimum on the order of $10^{-4}\mathrm{E_h}$.

To justify the σ truncation, it is crucial to quantify the energy scales present in the DMBE-PES relative to the thermal energy. The potential encompasses two distinct domains: as shown in Fig. S4 the van der Waals (vdW) domain (green line), where the well depth (4.31x$10^{-4}E_h$) corresponds to a thermal scale of approximately 136 K (which corresponds to the regime of thermodynamic validity); and the chemical bond formation domain, where the deep well (0.15 $\mathrm{E_h}$, black line) corresponds to a thermal energy scale exceeding 30.000 K. In this chemical domain, the condition $|E_{bound}| >> k_B T$ is met for all relevant temperatures, causing the Mayer function (f(r)) to diverge and thus invalidating the dilute gas model. Therefore, the σ truncation is not a numerical artifact, but a fundamental methodological and physical requirement to exclude the high-energy regimefrom the inte-

gral, thereby ensuring the thermodynamic validity of the virial expansion for the low-energy domain (around $10^{-4}E_h$).

This physical argument can be expressed mathematically as:

$$\lim_{k_BT \ll |E_{bound}|} e^{-u(r)/(k_BT)} - 1 \rightarrow \infty \tag{7}$$

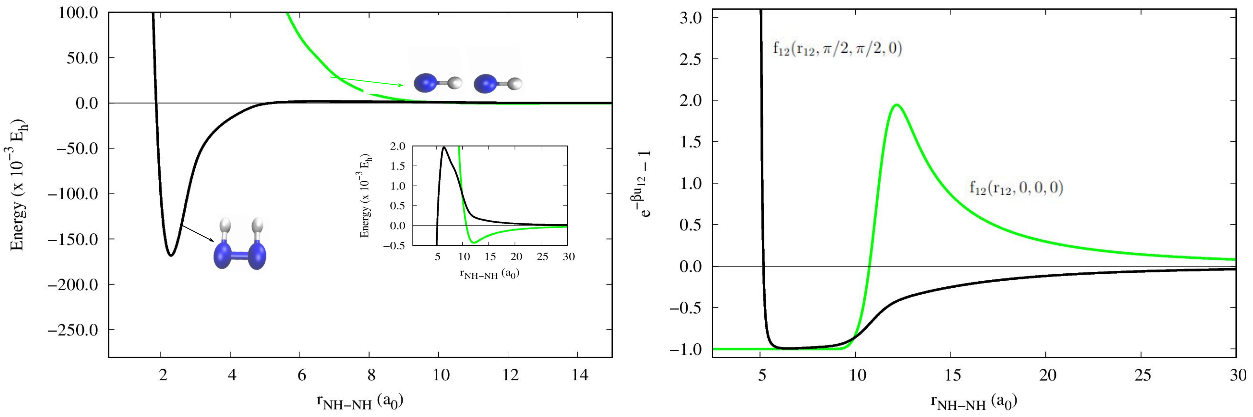

FIG. S4. $f_{12}(r_{12}, \theta_1, \theta_2, \phi) = \exp\big[-\beta u_{12}(r_{12}, \theta_1, \theta_2, \phi)\big] - 1$. Black curve corresponding to the geometry ($\theta_1 = \pi/2$, $\theta_2 = \pi/2$, $\phi = 0$). Green curve corresponds to the geometry ($\theta_1 = 0$, $\theta_2 = 0$, $\phi = 0$)

Coordinate Transformation in the NH Dimer

$R_{NH} = 1.965\ a_0$	N-H bond length
$r_1 = R_{NH} - \dfrac{m_H}{m_H + m_N} R_{NH}$	distance from H to center of mass
$r_2 = \dfrac{m_H}{m_H + m_N} R_{NH}$	distance from N to center of mass
$r_1 + r_2 = R_{NH}$	sum of partial distances equals bond length
$\vec{R}_{N_1} = \big(r_2 \cos\theta_1,\ -r_2 \cos\phi \sin\theta_1,\ -r_2 \sin\phi \sin\theta_1\big)$	position of N(1)
$\vec{R}_{N_2} = \big(r_{12} + r_2 \cos\theta_2,\ -r_2 \sin\theta_2,\ 0\big)$	position of N(2)
$\vec{R}_{H_4} = \big(r_{12} - r_1 \cos\theta_2,\ r_1 \sin\theta_2,\ 0\big)$	position of H(4)
$\vec{R}_{H_3} = \big(-r_1 \cos\theta_1,\ r_1 \cos\phi \sin\theta_1,\ r_1 \sin\phi \sin\theta_1\big)$	position of H(3)

Coordinate Transformation in the N_2-H_2 Dimer

$R_{NN} = 2.074\ a_0$	distance between N atoms
$R_{HH} = 1.401\ a_0$	distance between H atoms
$r_1 = \frac{1}{2} R_{HH}$	half of H-H bond length
$r_2 = \frac{1}{2} R_{NN}$	half of N-N bond length
$\vec{R}_{H_4} = \big(r_1 \cos\theta_1,\ -r_1 \cos\phi \sin\theta_1,\ -r_1 \sin\phi \sin\theta_1\big)$	H(4) position relative to H-H center of mass
$\vec{R}_{H_3} = \big(-r_1 \cos\theta_1,\ r_1 \cos\phi \sin\theta_1,\ r_1 \sin\phi \sin\theta_1\big)$	H(3) position relative to H-H center of mass
$\vec{R}_{N_2} = \big(r_{12} - r_2 \cos\theta_2,\ r_2 \sin\theta_2,\ 0\big)$	N(2) position relative to N-N center of mass
$\vec{R}_{N_1} = \big(r_{12} + r_2 \cos\theta_2,\ -r_2 \sin\theta_2,\ 0\big)$	N(1) position relative to N-N center of mass

S2. TABLES FOR SECOND VIRIAL COEFFICIENTS

TABLE S1: Interaction second virial coefficient in terms of the temperature for $N_2 \cdots H_2$ This table shows how the parameter σ, defined in Eq. 4 , influences the value of B(T). The values of Ref. 1 are experimental data, and the superscript "b" refers to values obtained through the polynomial form $B_{12}/\mathrm{cm^3 mol^{-1}} = 3.5658 \cdot 10 - 7.4422 \cdot 10^3/(T/K) + 1.4167 \cdot 10^5/(T/K)^2 - 7.5396 \cdot 10^6/(T/K)^3$.

**T/K**	σ/a_0					References		
	5.4	5.5	5.6	5.7	5.8	(1)	(4)	(2)
30	-411.162	-380.498	-360.327	-353.694	-357.838	—	—	-208.084
35	-270.440	-254.718	-244.867	-241.879	-243.912	—	—	-168.644
36	-251.650	-237.683	-229.040	-226.503	-228.372	-227.0(2.0)	—	-162.233
40	-194.103	-185.094	-179.851	-178.639	-180.200	-184.0(2.0)	-218.163	-140.160
44	-155.091	-149.047	-145.834	-145.469	-146.994	-150.0(2.0)	—	-122.523
45	-147.263	-141.772	-138.937	-138.737	-140.272	—	—	-118.656
48	-127.144	-123.008	-121.100	-121.316	-122.903	-123.0(1.0)	—	-108.117
50	-115.991	-112.564	-111.140	-111.582	-113.215	$-116.8(5)^{b}$	-149.558	-101.862
52	-106.262	-103.428	-102.410	-103.047	-104.727	-106.0(1.0)	—	-96.133
55	-93.811	-91.700	-91.178	-92.059	-93.813	—	—	-88.392
56	-90.138	-88.233	-87.852	-88.805	-90.583	-95.0(1.0)	—	-86.012
60	-77.353	-76.139	-76.232	-77.430	-79.297	-92.0(1.0)	-114.051	-77.353
65	-64.712	-64.141	-64.676	-66.110	-68.074	—	—	-68.145
70	-54.734	-54.643	-55.508	-57.122	-59.164	—	—	-60.348
75	-46.684	-46.963	-48.081	-49.835	-51.939	$-56.3(3)^{b}$	—	-53.663
80	-40.072	-40.643	-41.959	-43.823	-45.975	—	—	-47.869
85	-34.558	-35.366	-36.840	-38.791	-40.978	—	—	-42.798
90	-29.902	-30.903	-32.507	-34.526	-36.738	—	—	-38.325
95	-25.927	-27.089	-28.798	-30.871	-33.100	—	—	-34.349
100	-22.502	-23.800	-25.595	-27.710	-29.948	$-32.1(3)^{b}$	-37.608	-30.792
105	-19.527	-20.939	-22.805	-24.953	-27.194	—	—	-27.591

continues on the next page

TABLE S1 – *table continuation*

**T/K**	σ/a_0					References		
	5.4	5.5	5.6	5.7	5.8	(1)	(4)	(2)
110	-16.923	-18.433	-20.358	-22.530	-24.770	—	—	-24.696
115	-14.630	-16.224	-18.198	-20.388	-22.622	—	—	-22.065
120	-12.600	-14.265	-16.279	-18.482	-20.708	—	—	-19.663
125	-10.792	-12.519	-14.567	-16.777	-18.991	—	—	-17.462
130	-9.176	-10.956	-13.030	-15.244	-17.445	—	—	-15.438
135	-7.724	-9.551	-11.646	-13.860	-16.046	—	—	-13.569
140	-6.415	-8.282	-10.395	-12.606	-14.775	—	—	-11.840
145	-5.231	-7.132	-9.258	-11.464	-13.616	—	—	-10.234
148.15	-4.574	-6.494	-8.626	-10.828	-12.969	-10.3(3.0)	—	-9.280
150	-4.156	-6.087	-8.223	-10.422	-12.555	-9.9(3)[b]	-8.899	-8.739
170	-0.717	-2.730	-5.612	-7.036	-9.090	-5.0(0.3)	-4.249	-3.669
173.15	-0.297	-2.318	-4.465	-6.615	-8.657	-3.4(3.0)	—	-2.981
180	0.606	-1.430	-3.573	-5.704	-7.717	-2.6(0.2)	—	-1.570
190	1.734	-0.317	-2.450	-4.551	-6.524	-0.6 (0.1)	—	0.300
198.15	2.520	0.462	-1.660	-3.737	-5.679	1.2(3.0)	—	1.681
200	2.236	0.642	-1.945	-3.547	-5.480	1.0(3)[b]	0.899	1.979
203.15	2.965	0.905	-1.209	-3.271	-5.192	5.7(3.0)	—	2.473
210	3.537	1.476	-0.626	-2.666	-4.561	2.7(0.1)	—	3.494
220	4.262	2.202	0.120	-1.888	-3.745	4.1(0.2)	—	4.867
223.15	4.461	2.402	0.326	-1.672	-3.519	4.8(3.0)	—	5.273
230	4.895	2.840	0.779	-1.197	-3.019	5.3(0.2)	—	6.118
240	5.449	3.403	1.364	-0.581	-2.368	6.2(0.2)	—	7.262
248.15	5.844	3.806	1.785	-0.135	-1.895	8.0(3.0)	—	8.124
250	5.936	3.900	1.885	-0.029	-1.782	7.2(0.2)	8.307	8.312
260	6.367	4.343	2.352	0.468	-1.254	8.2(0.2)	—	9.280
270	6.748	4.738	2.770	0.916	-0.775	9.1(0.2)	10.788	10.175

continues on the next page

TABLE S1 – *table continuation*

T/K	σ/a_0					References		
	5.4	5.5	5.6	5.7	5.8	(1)	(4)	(2)
273.15	6.854	4.849	2.888	1.042	-0.641	10.5(0.5)	—	10.443
275	6.923	4.920	2.965	1.124	-0.553	10.8 (0.4)	—	10.597
290	7.389	5.410	3.490	1.692	0.056	12.0 (0.4)	—	11.775
293.15	7.473	5.499	3.586	1.796	0.168	11.7(0.5)	—	12.007
298.15	7.606	5.641	3.740	1.964	0.348	13.0(3.0)	—	12.364
300	7.657	5.696	3.800	2.028	0.418	12.1(3)[b]	12.725	12.494
310.15	7.899	5.954	4.082	2.336	0.750	13.3(0.4)	—	13.175
313.15	7.966	6.027	4.161	2.423	0.906	13.1(0.5)	—	13.367
323.15	8.175	6.253	4.411	2.697	1.141	14.6(3.0)	—	13.983
330	8.308	6.399	4.573	2.876	1.335	14.6(0.4)	—	14.383
333.15	8.363	6.459	4.639	2.949	1.415	14.8(0.5)	—	14.562
350	8.639	6.766	4.983	3.331	1.831	15.4(0.4)	—	15.460
400	9.219	7.434	5.752	4.198	2.783	—	—	17.674
450	9.564	7.862	6.266	4.792	3.443	—	—	19.390
500	9.765	8.137	6.614	5.206	3.909	—	—	20.758
550	9.872	8.310	6.850	5.495	4.240	—	—	21.875
600	9.917	8.413	7.006	5.694	4.474	—	—	22.803
650	9.918	8.466	7.103	5.829	4.637	—	—	23.588
700	9.890	8.482	7.158	5.916	4.748	—	—	24.260
750	9.839	8.471	7.181	5.966	4.819	—	—	24.841
800	9.773	8.440	7.179	5.988	4.860	—	—	25.349
850	9.696	8.393	7.159	5.988	4.877	—	—	25.797
900	9.610	8.335	7.124	5.972	4.877	—	—	26.195
950	9.519	8.269	7.078	5.943	4.863	—	—	26.550
1000	9.423	8.195	7.023	5.904	4.838	—	—	26.870
1500	8.408	7.326	6.283	5.285	4.337	—	—	28.891

continues on the next page

TABLE S1 – *table continuation*

T/K	σ/a_0					References		
	5.4	5.5	5.6	5.7	5.8	(1)	(4)	(2)
2000	7.490	6.497	5.544	4.639	3.790	—	—	29.899
RMSD	7.383	6.943	7.415	7.752	8.048	—	—	—
c.c	0.995	0.996	0.997	0.997	0.997	—	—	—

end of table

TABLE S2. Second virial coefficient over a temperature range between 290 K and 350 K. Parameter σ fixed in 5.0 a_0,5.1 a_0 and 5.2 a_0 presents a better degree of representativeness for B(T) when compared with other values according to table S1.

T/K	σ/a_0					References		
	5.4	5.5	5.6	5.7	5.8	(1)	(4)	(2)
290	14.319	12.874	11.200	9.350	7.389	12.0 (0.4)	—	11.775
293	14.420	12.960	11.290	9.430	7.473	11.7(0.5)	—	12.007
298	14.580	13.110	11.420	9.560	7.606	13.0(3.0)	—	12.364
300	14.638	13.163	11.450	9.590	7.657	12.1(3)[b]	12.725	12.494
310	14.920	13.418	11.710	9.840	7.899	13.3(0.4)	—	13.175
313	15.000	13.490	11.770	9.910	7.966	13.1(0.5)	—	13.367
323	15.240	13.700	11.970	10.110	8.175	14.6(3.0)	—	13.983
330	15.387	13.839	12.100	10.230	8.308	14.6(0.4)	—	14.383
333	15.450	13.890	12.150	10.290	8.363	14.8(0.5)	—	14.562
350	15.749	14.163	12.410	10.540	8.639	15.4(0.4)	—	15.460

S3. VALIDATION OF THE CALCULATION

The inspection of Figures S3, S4, and table S1 shows that B^{cal} at 40 K and 50 K temperatures present relative errors of 0.59% and 0.69%, respectively, while the theoretical work of Ref. 2 presented relative errors of 23.83% and 12.79% compared to $B^{exp}(T)$[1]. For temperatures between 130 and 350 K, the second virial coefficient was calculated with errors less than 15 cm^3mol^{-1}. From the graphs presented in Figure S4, the best value of σ to reproduce the experimental value of B(T) changes with temperature. Thus, fixing the same value of σ for all temperatures is not a good choice.

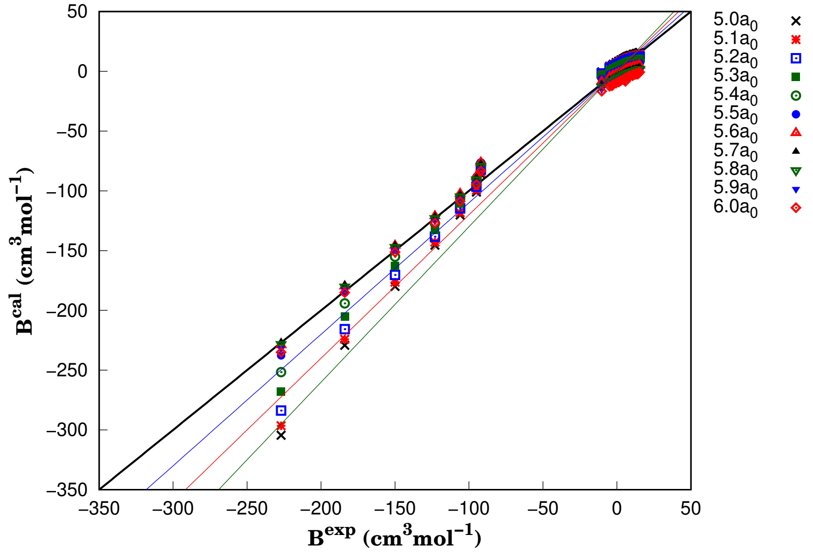

FIG. S5. Scatter plot relates the values $B^{cal}(T)$ and $B^{exp}(T)$ from Ref. 1. The black, blue, red, and green lines represent relative errors of 0%, 10%, 20%, and 30%, respectively. This analysis is more consistent for negative values of B(T).

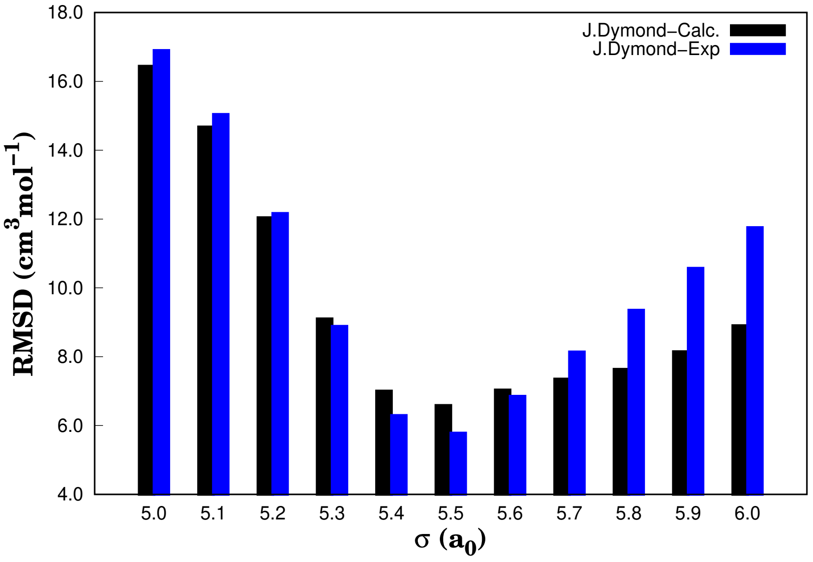

FIG. S6. RMSD obtained for the second virial coefficient calculated by Eq. 4 using DMBE-PES to several σ values. The RMSD is calculated relative to experimental and theoretical data collected from the reference 1 $N_2 \cdots H_2$

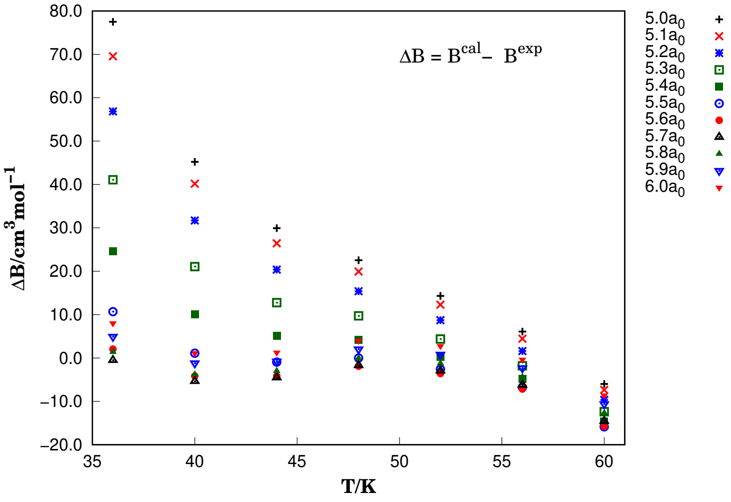

FIG. S7. Deviation of calculated results for second virial coefficient to $N_2 \cdots H_2$ for temperatures between 36 K and 60 K, or different values of σ. All errors are calculated relative to the experimental data from Ref. 1.

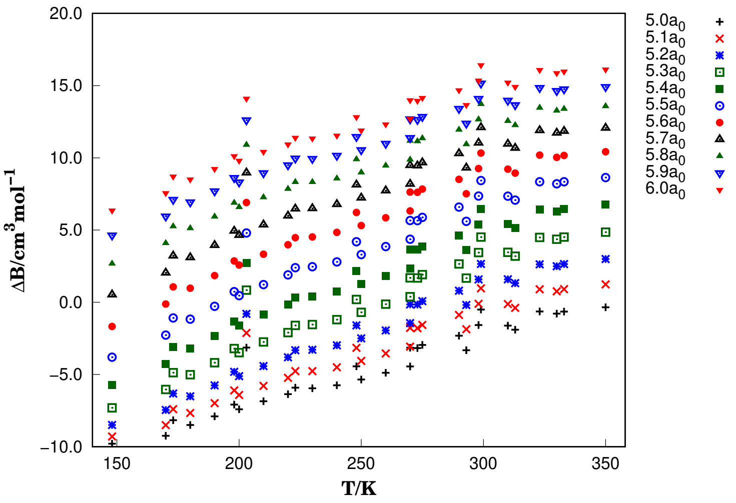

FIG. S8. Deviation of calculated results for second virial coefficient to $N_2 \cdots H_2$ for temperatures between 148.15 K and 350 K for different values of σ. All errors are calculated relative to the experimental data of reference 1.

TABLE S3. Root-mean-square deviations (RMSD) of calculated B(T) in cm^3mol^{-1}, relatively to the experimental (a) and theoretical (b) data from Ref. 1, for different values of σ.

σ/a_0	5.0	5.1	5.2	5.3	5.4	5.5	5.6	5.7	5.8	5.9	6.0
RMSD^a	16.900	15.040	12.165	8.883	6.295	5.781	6.852	8.140	9.354	10.570	11.752
RMSD^b	17.332	15.470	12.694	9.595	7.383	6.943	7.415	7.752	8.048	8.588	9.386
c.c	0.993	0.994	0.994	0.994	0.995	0.996	0.997	0.997	0.997	0.997	0.997

S4. NH···NH

The values of B(T) calculated for values of σ between 4.7 and 4.8 a_0 as shown in Fig. 3 of the paper. With these values of σ, we will construct a confidence interval for B(T) as a function of T. However, in calculations of thermodynamic properties, we will use the value $\sigma = 4.7\ a_0$. The virial coefficients obtained were adjusted to fit the data using the following analytical expression:

$$\mathrm{B}(T) = \sum_{i=0}^{3} b_i \left(\frac{T_0}{T} - 1\right)^i$$

TABLE S4. Following a similar procedure as for the $N_2 \cdots H_2$ system, the value of the second virial coefficient B(T) for the NH $\cdots$ NH system, obtained from the methodology proposed in this work, and the fitting form can be represented as: $B(T) = \sum_{i=0}^{3} b_i \left(\frac{T_0}{T} - 1\right)^i$. In these cases, the temperatures T are in Kelvin, and $T_0 = 298.15\,K$. The dimensionless parameter are $b_i^* = b_i/10^2\ cm^3\ mol^{-1}$

σ/a_0	b_0^*	b_1^*	b_2^*	b_3^*
4.7	−5.02475	−16.6485	−19.3327	−8.01048
4.8	−3.09957	−8.2962	−7.3354	−2.34555

TABLE S5. Second virial coefficient B(T) against temperature for NH $\cdots$ NH dimer. Values of B(T) predicted by Eq. 4. for several values of σ.

**T/K**	$\sigma = 4.7a_0$	$\sigma = 4.8a_0$	**T/K**	$\sigma = 4.7a_0$	$\sigma = 4.8a_0$	**T/K**	$\sigma = 4.7a_0$	$\sigma = 4.8a_0$
200	-11098.7	-3011.286	1050	-6.944	-5.983	2100	11.796	11.636
250	-1668.41	-703.280	1100	-4.690	-3.884	2200	12.256	12.069
300	-475.200	-320.278	1150	-2.716	-2.040	2300	12.635	12.424
350	-284.043	-196.168	1200	-0.980	-0.413	2400	12.947	12.715
400	-179.214	-137.449	1250	0.554	1.0280	2500	13.202	12.952
450	-126.357	-103.026	1300	1.916	2.308	2600	13.409	13.142
500	-94.708	-80.166	1350	3.128	3.450	2700	13.575	13.294
550	-73.566	-63.776	1400	4.211	4.471	2800	13.706	13.412
600	-58.390	-51.420	1450	5.181	5.387	2900	13.807	13.502
650	-46.946	-41.773	1500	6.051	6.209	3000	13.882	13.568
700	-38.008	-34.045	1550	6.834	6.949	3500	13.983	13.634
750	-30.843	-27.731	1600	7.539	7.616	4000	13.807	13.437
800	-24.983	-22.493	1650	8.176	8.218	4500	13.496	13.115
850	-20.113	-18.091	1700	8.752	8.763	5000	13.121	12.735
900	-16.014	-14.353	1800	9.746	9.703	5500	12.722	12.333
950	-12.526	-11.150	1900	10.564	10.475	6000	12.318	11.931
1000	-9.533	-8.386	2000	11.239	11.112	7000	11.539	11.157

S5. MAPPING OF THE LONG-RANGE INTEGRAL AND DEPENDENCE ON σ

In our work, we employed the transformation

$$r = \sigma + \frac{x}{1-x}, \quad x \in [0,1), \tag{8}$$

to map the long-range part of the integral from the semi-infinite domain $[\sigma, \infty)$ onto the finite interval $[0, 1)$.

1. Verification of the Mapping

We can verify the limits of the transformation:

- As $x \to 0$:
$$r = \sigma + \frac{0}{1-0} = \sigma$$
- As $x \to 1^-$:
$$r = \sigma + \frac{1^-}{1-1^-} \to \sigma + \infty = \infty$$

Thus, the transformation correctly maps $x \in [0, 1)$ to $r \in [\sigma, \infty)$.

2. Transformation of the Integral

For the second virial coefficient, the Mayer function integral is typically written as

$$B = -2\pi \int_{\sigma}^{\infty} r^2 f(r)\, dr. \tag{9}$$

Applying the variable change $r = \sigma + \frac{x}{1-x}$, we have

$$dr = \frac{d}{dx}\left(\sigma + \frac{x}{1-x}\right) dx = \frac{dx}{(1-x)^2}, \tag{10}$$

and the integral becomes

$$B = -2\pi \int_0^1 \left(\sigma + \frac{x}{1-x}\right)^2 f\left(\sigma + \frac{x}{1-x}\right) \frac{dx}{(1-x)^2}. \tag{11}$$

3. Dependence on σ

Although the integral is now defined on a finite domain, σ explicitly appears in the integrand, as $\sigma + \frac{x}{1-x}$. For strongly repulsive or anisotropic potentials, the integral remains highly sensitive to σ, and small variations in σ can significantly affect the calculated value of $B(T)$. Therefore, this transformation does not remove the physical dependence on σ, but it provides a convenient mapping for numerical evaluation of the semi-infinite integral.

---

[1] JD Dymond, KN Marsh, RC Wilhoit, and KC Wong. *Virial coefficients of pure gases and mixtures*, volume 21. Springer, 2003.

[2] JF Estela-Uribe, J Jaramillo, MA Salazar, and JPM Trusler. Virial equation of state for natural gas systems. *Fluid phase equilibria*, 204(2):169–182, 2003.

[3] LA Poveda, M Biczysko, and AJC Varandas. Accurate ab initio based dmbe potential energy surface for the ground electronic state of n 2 h 2. *THE JOURNAL OF CHEMICAL PHYSICS*, 131:044309, 2009.

[4] Phąm Vǎn Tat and Ulrich K Deiters. Calculation of cross second virial coefficients using ab initio intermolecular potential energy surfaces for dimer $H_2 - -N_2$. *Chemical Physics*, 517:208–221, 2019.
